# Identification of Phenolic Compounds from *Hancornia speciosa* (Apocynaceae) Leaves by UHPLC Orbitrap-HRMS

**DOI:** 10.3390/molecules22010143

**Published:** 2017-01-15

**Authors:** Katherine Xavier Bastos, Clarice Noleto Dias, Yuri Mangueira Nascimento, Marcelo Sobral da Silva, Silvana Maria Zucolotto Langassner, Ludger A. Wessjohann, Josean Fechine Tavares

**Affiliations:** 1 Departamento de Ciências Farmacêuticas, Universidade Federal da Paraíba, João Pessoa 58051-900, Brazil; katherine_xb@hotmail.com (K.X.B.); clarice@ltf.ufpb.br (C.N.D.); yurimangueira@ltf.ufpb.br (Y.M.N.); marcelosobral@ltf.ufpb.br (M.S.d.S.); 2 Dept. Bioorganic Chemistry, Leibniz Institute of Plant Biochemistry, Weinberg 3, Halle 06120, Germany; wessjohann@ipb-halle.de; 3 Departamento de Farmácia, Universidade Federal do Rio Grande do Norte, Natal 59010-180, Brazil; silvanazucolotto@ufrnet.br

**Keywords:** *Hancornia speciosa*, mangabeira, UHPLC, Orbitrap, chemical composition, phenolic compounds

## Abstract

Apocynaceae is a botanical family distributed mainly in tropical and subtropical regions of the world. In Brazil, they comprise about 90 genera and 850 species, inhabiting various types of vegetation. Within this large botanical family, the genus *Hancornia* is considered monotypic, with its only species *Hancornia speciosa* Gomes. Antihypertensive, antidiabetic, and antiviral activities are described for this species. Despite having been the target of some studies, knowledge of its chemical composition is still limited. In this study, the phenolics of *H. speciosa* leaves were analyzed using ultra-high performance liquid chromatography (UHPLC) coupled to Orbitrap high-resolution mass spectrometry (HRMS). As a result, 14 compounds were identified viz. protocatechuic acid, catechin, and quercetin, and another 14 were putatively identified viz. B- and C-type procyanidins, while just one compound remained unknown. From the identified compounds, 17 are reported for the first time viz. coumaroylquinic acid isomers and eriodyctiol. The results show that *Hancornia speciosa* can serve as source of valuable phenolics.

## 1. Introduction

Apocynaceae is a botanical family that includes between 3700 and 5100 species, which are distributed almost all over the world, but mainly in tropical and subtropical regions. Approximately 90 genera and 850 species of Apocynaceae occur in Brazil, inhabiting various types of vegetation. Within this large botanical family is the monotypic genus *Hancornia*, with the species *Hancornia speciosa* Gomes [1].

Popularly known as “mangabeira”, *H. speciosa* is a native Brazilian fruit tree that is distributed from the north to the south of the country. The phytogeographic domains are the Amazon Rainforest (mainly Pará and Amapá States), Caatinga, Central Brazilian Savanna (cerrado), and Atlantic Rainforest [2]. The fruit name “mangaba” comes from the indigenous language Tupi-guarani and means “good fruit for eating”. The tree is medium-sized (2 to 10 m tall) with an irregular canopy and a twisted, highly-branched trunk. The branches are smooth and reddish, exuding latex over its entire length [3,4]. The flowering of *H. speciosa* occurs from August to November, and the fruits ripen between September and November in the cerrado [5].The fruits are berry type, varying in size, shape, and color, and are generally ellipsoidal and yellowish or greenish in color. They are edible, and are a rich source of vitamin C [6]. Sustainable harvesting of these fruits by local communities is of great economic and social importance. The fruits are usually consumed in natura, or processed in industries for ice cream, jam, and juice preparation due to their pleasant flavor and nutritional properties [7,8].

Ethnobotanical surveys show the traditional use of *H. speciosa* by some communities in Brazil. Preparations with different parts of the tree have been used for treating wounds, as anti-inflammatory, antirheumatic, antihypertensive, anti-obesity, and antidiabetic products, and to prevent gastric lesions [9,10,11,12].

Some pharmacological activities have already been described for the leaves of *H. speciosa* in the scientific literature. The ethanolic extract was shown to possess a potential antihypertensive effect on the basis of a reduction in peripheral resistance induced by inhibiting angiotensin I-converting enzyme, production of nitric oxide, and a mechanism involving increased production of H_2_O_2_ in the mesenteric arteries of hypertensive mice [13,14]. A potential antidiabetic effect has been claimed based on the inhibition of α-glucosidase and increased glucose uptake by adipocytes [15]. Other activities reported for *H. speciosa* leaves are acetylcholinesterase inhibition and antioxidant properties [16], wound healing, and anti-inflammatory [17] and antiviral activity [18].

Previous phytochemical studies have reported the identification of different classes of compounds in *H. speciosa* extracts. Chlorogenic acid, naringenin-7-*O*-glucoside, catechin, and proanthocyanidins were identified from the trunk latex [19]. The chemical composition of leaf extracts includes the following: cyclitols, such as bornesitol, quinic, and chlorogenic acids; flavonoids, such as rutin, kaempferol diglycoside, and kaempferol triglycoside; and triterpenes and steroids, such as α- and β-amirin, lupeol, β-sitosterol, obtusalin, and erythrodiol [15,19,20,21].

Despite having been the target of the mentioned studies, a comprehensive knowledge of the chemical composition of *H. speciosa* is limited. Therefore, in this study, an unbiased survey of phenolic constituents identifiable in the leaf extracts was envisaged by using ultra-high performance liquid chromatography (UHPLC) coupled to Orbitrap high-resolution mass spectrometry (HRMS).

## 2. Results

**Figure 1:**
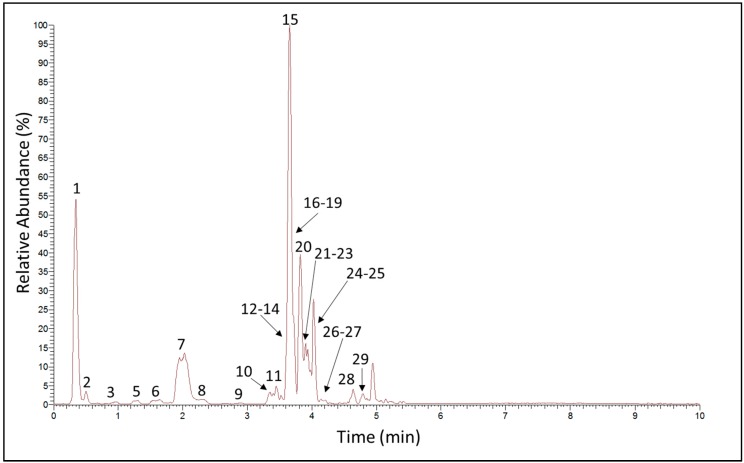
Negative base peak trace from ultra-high performance liquid chromatography–high-resolution mass spectrometry (UHPLC-HRMS) analysis of *Hancornia speciosa* ethanolic leaf extract.

**Figure 2:**
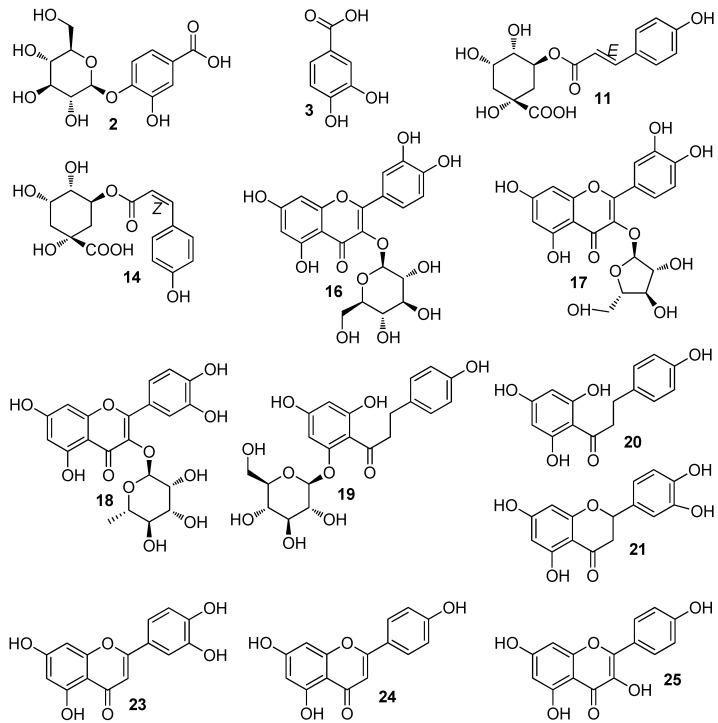
Chemical structures of the phenolic compounds identified for the first time in *Hancornia speciosa* leaf extract.

The constituents of the ethanolic extracts of *H. speciosa* were determined by UHPLC/MS/MS. Ethanol was selected as the extractive solvent because the previously described pharmacological activities were based on an ethanolic extract [13,14,15]. In a pilot study using an ion-trap machine, both positive and negative modes were analyzed. Visualization of both chromatograms revealed more intense and well-resolved chromatographic peaks in the negative compared to the positive ion mode. This is in line with the observation that *H. speciosa* is a species rich in phenolic compounds [19], which are supposed to ionize better and have a characteristic mass spectral behavior in the negative mode [22]. Therefore, profiling of the phenolics present in the ethanolic extract of *H. speciosa* leaf, negative electrospray ionization (ESI) mode with a UHPLC run of 15 min was adopted as method of choice. Figure 1 shows the base peak chromatogram obtained under conditions described in detail in the Materials and Methods section. Compounds were identified through co-injection with reference samples or on the basis of fragmentation patterns compared with literature data. Thus, it was possible to identify 28 compounds, while one compound remained unknown. Table 1 compiles the data of the suggested compounds. Chemical structures of these compounds are given in Figure 2. Out of the 28 identified compounds, 15 are described here for the first time for *H. speciosa*.

Compound **1**, eluted at 0.35 min, was identified on the basis of the ion obtained [M − H]^−^ at *m/z* 191.0559. Literature ascribes this exact mass to the structure of quinic acid [23]. This compound was previously reported in the leaves of *H. speciosa* [15]. Compound **2** at 0.68 min showed a mass spectrum with a [M − H]^−^ peak at *m/z* 315.0716. Examination of mass data banks showed these data to be consistent with the structure of protocatechuic acid hexoside. This finding was reinforced by the analysis of the fragments formed from this ion at *m/z* 153 originating from hydrolysis with the loss of an osidic moiety of 109 mass units, giving rise to the fragment *m/z* 153 after loss of CO_2_, which is in line with literature data for the suggested structure [24]. This compound is reported for the first time in *H. speciosa*.

Compound **3** at 0.85 min showed a mass spectrum with the [M − H]^−^ peak at *m/z* 153.0193, consistent with the structure of protocatechuic acid. This finding was reinforced by co-injection of a standard sample of this compound, which is reported for the first time in *H. speciosa*. At least two compounds co-eluted at 1.30 min; the ion at *m/z* 343.1027 did not match any compound listed in the databases (compound **4**), and the other at *m/z* 577.1341 was consistent with the deprotonated molecule of B-type procyanidin (compound **5**). Likewise, the mass spectra from the peaks at 2.88 and 3.56 min had similar molecular ions and fragmentation patterns as compound **5**, which led us to believe that compounds **9** and **13** were also B-type procyanidins. These compounds have already been reported in infusions of the bark of *H*. *speciosa* by direct injection [25].

The peaks at 1.64 and 3.36 min showed a molecular ion at *m/z* 289.0712, and were assigned to the deprotonated molecule of (+)-catechin (compound **6**) and (−)-epicatechin (compound **10**), respectively, according to the retention time of their corresponding standards. For both molecules, the main fragment produced in the MS^2^ spectra was the [M – H − CO_2_]^−^ ion (*m/z* 245). This dominant fragmentation behavior when using Orbitrap has been previously described [22]. In addition, MS^2^ spectra revealed two other fragments, *m/z* 205 generated from a scission in the B ring, and *m/z* 179 from the loss of a catechol moiety.

Compound **7**, eluted at 2.03 min, showed a molecular ion peak at *m/z* 353.0873. The fragmentation of this peak showed an ion at *m/z* 191 [M − 162 − H]^−^, due to the loss of a caffeoyl moiety, and further peaks at *m/z* 233, 179, and 173, which are characteristic fragments of chlorogenic acid. The co-injection with this reference compound confirmed its identification. At 2.17 min, the ion peak at *m/z* 179.0348 was assigned to the deprotonated molecule of caffeic acid (compound **8**).

The peaks at 3.45 and 3.62 min showed a deprotonated molecule at *m/z* 337.0923, and were tentatively assigned to coumaroylquinic acid isomers (compounds **11** and **14**). This finding was supported by its product ion spectrum, which showed peaks at *m/z* 191, 173, and 163, obtained through heterolytic scissions and loss of water, which is in agreement with literature data [26]. These isomers are reported for the first time for *H. speciosa*.

Compound **12** at 3.53 min showed a molecular ion peak at *m/z* 865.1958, which was assigned to a C-type procyanidin. Additionally, this deprotonated molecule showed a fragment ion at *m/z* 577 due to a homolytic scission, corresponding to the dimer unit, and at *m/z* 695, originating from a retro-Diels–Alder fragmentation and loss of water. These data corroborate the literature data for the suggested chemical structure [25]. This is the first report of a C-type procyanidin in an *H. speciosa* extract.

The peak at 3.65 min had a molecular ion at *m/z* 609.1453, and was assigned to rutin (compound **15**). This identification was supported by the base peak in its MS^2^ spectrum (*m/z* 301), corresponding to the loss of a rutinoside moiety, and on the basis of the co-injection of a reference compound. Rutin has already been reported in *H. speciosa* leaves [15]. Compound **16** at 3.70 min showed a mass spectrum with a molecular peak at *m/z* 463.0887, consistent with the structure of quercetin hexoside. Like rutin, the deprotonated molecule also had a product ion at *m/z* 301 due to the loss of a hexose moiety. Quercetin 3-β-d-glucoside standard was co-injected, and had a similar retention time. Hence, we can suggest that the hexose moiety was bound to position C3 of quercetin. Following the elution order, compounds **17** and **18** were tentatively assigned as quercetin pentoside ([M − H]^−^ at *m/z* 433.0770) and quercetin-3-*O*-rhamnoside ([M − H]^−^ at *m/z* 447.0927), respectively. The fragmentation patterns of both deprotonated molecules were consistent with quercetin derivatives described in the literature, showing a base peak at *m/z* 301 [27,28]. Compounds **17** and **18** are reported for the first time for *H. speciosa*.

The glycosylated chalcone phlorizin (compound **19**) was identified in the studied extract with a retention time of 3.80 min. Several peaks with retention times between 3.82 and 4.06—showing molecular ions at *m/z* 273.0764, 287.0559, 301.0551, 285.0402, 269.0450, and 285.0401—were identified as phloretin (compound **20**), eriodyctiol (compound **21**), quercetin (compound **22**), luteolin (compound **23**), apigenin (compound **24**), and kaempferol (compound **25**), respectively. Compounds **19**–**25** were identified on the basis of their mass spectrum and the co-injection of standard compounds. With exception of compound **22**, the others are reported for the first time for *H. speciosa*.

Four hydroxylated fatty acids (compounds **26**–**29**) eluting late in the chromatogram (Rt 4.08–4.75 min) were annotated based on their respective molecular ion masses. Peaks **26** (*m/z* 327.2172) and **27** (*m/z* 329.2326) were assigned as trihydroxy-octadecadienoic and trihydroxy-octadecenoic acid, respectively. A peak eluting at 4.59 min characterized dihydroxy-octadecadienoic acid (**28**) ([M − H]^−^ at *m/z* 311.2221). Peak 29 was tentatively identified as hydroxy-octadecatrienoic acid, with [M − H]^−^ at *m/z* 293.2116 and product ions at *m/z* 275 and 249 corresponding to the loss of water molecule (−18 Da) and a carboxyl group (−44 Da), respectively. It is worth mentioning that hydroxy fatty acids were not previously described in *H. speciosa* extracts.

## 3. Discussion

The present work reports a detailed chemical characterization of the ethanolic extract of *H. speciosa* leaves, using UHPLC coupled to HRMS in negative mode. Phenolic compounds comprise the majority in the composition of the studied plant, and these compounds are probably correlated to some of the pharmacological activities previously described in the literature. Our finding of phloretin and phlorizin can especially be seen as support for the proposed antidiabetic activity of the species. These compounds are SGLT1/2 inhibitors, and are found in apples or other health foods. They are suggested to influence glucose perception and signaling in humans [29]. In addition to phloretin/phlorizin, the flavonols quercetin, kaempferol, and rutin—identified in *H. speciosa* extract—can play a role in the serum glucose balance. The aglycone flavonoids reduce the activity of the duodenal maltase [30], while rutin beyond that is able to retard deterioration from symptomatic diabetes to death and stabilize body-weight [31]. Altogether—and in addition to the presence of cyclitols (see above)—the presence of these phenolics in *H. speciosa* can be seen as support for the proposed antidiabetic activity of this plant and its potential use for treatment of diabetes mellitus.

The previously reported antihypertensive effect of *H. speciosa* ethanolic leaf extract [14,32] can be explained by the high level of phenolics in this type of extract, as shown in the present study. The intake of this class of secondary metabolites can contribute to the treatment of hypertension via the stimulation of several mechanisms of action that reduce blood pressure [33,34]. More specifically, rutin—the major peak in the HPLC fingerprint of this extract—was shown to contribute to the antihypertensive property of *H. speciosa* by the induction of endothelium-dependent vasodilation in rat superior mesenteric arteries [35]. Similar to the chemical composition of the leaves shown herein, the aqueous fruit extract of the same species showed the phenolics rutin and chlorogenic acid as prominent constituents. The fruit extract and the trunk latex exhibited anti-inflammatory activity in animal models [36,37]. Additionally, the latex showed significant angiogenic activity and a potential osteogenic effect, and had no cytotoxic or genotoxic effects on life systems [19,38,39]. Chemical analysis of *H. speciosa* latex resulted in the identification of naringenin-7-*O*-glucoside, chlorogenic acid, catechin, and procyanidin [19]. The ethanolic extract of *H. speciosa* bark was efficient in the prevention of gastric ulcers in rodents, together with anti-*Helicobacter pylori* effects [10]. It may be speculated that the here-described hydroxyl fatty acids for which antibiotic behavior is reported further contribute to this activity [40].

The fruit pulp of *H. speciosa* is rich in minerals, viz. potassium, copper, zinc, and iron; ascorbic acid and phenolics, viz. catechin, rutin, chlorogenic, vanillic, coumaric, and rosmarinic acids. The absence of toxic effects in in vivo assays—in addition to the antimutagenic properties of this fruit—highlight its potential as a functional food [41]. All of these findings support the widespread applicability of *H. speciosa* in the food and pharmaceutical industry to produce nutraceuticals and herbal drugs for varied treatments.

In the present work, 28 compounds were detected via UHPLC-HRMS in *H. speciosa* ethanolic extract. Phenolics such as rutin (**15**), quinic (**1**), and chlorogenic (**7**) acids have been widely reported in the bark and leaf extracts of this species [20,21,25]. A direct flow injection into the ESI source of a mass analyzer of the bark infusion of *H. speciosa* led to the tentative identification of (epi)catechin and procyanidins (dimers to hexamers of (epi)catechin) [25]. Herein, (+)-catechin (**6**) and (−)-catechin (**10**) could be confirmed in the leaf extract because of the use of a separation step (UHPLC) and the co-injection of reference standards, which were not included in the aforementioned method. Procyanidins viz. dimers (**5**, **9**, and **13**) and a trimer (**12**) of (epi)catechin could be annotated in the ethanolic leaf extract in the same way as they were in the bark infusion study [25]. Derivatives of caffeic acid, quercetin, and kaempferol are commonly found in *H. speciosa* leaf extracts [15,42]. The identification of their aglycone forms in the present work was supported by the co-injection of reference standards.

Although phenolics are widely identified in species belonging to the Apocynaceae family [43,44], most of its genera is known to have monoterpene indole alkaloids as the major class of secondary metabolites [45,46,47,48]. *Hancornia* seems to be an exception; no alkaloids have been described for this species. Despite these phytochemical differences, *H. speciosa* and other Apocynaceae plants share some pharmacological activities—e.g., antidiabetic [49], antibacterial [50], cytotoxic [51], and anti-inflammatory [52] properties.

The flavones luteolin (**23**) and apigenin (**24**)—reported for the first time in *H. speciosa*—and the flavonol quercetin (22) are some of the most efficacious plant flavonoids for cancer chemoprevention [53]. In general, plants rich in phenolic acids and flavonoids show a broad range of therapeutic properties that can contribute to a reduction in the incidence of chronic health problems, such as cancers, diabetes, and cardiovascular diseases [54,55,56].

## 4. Materials and Methods

### 4.1. Plant Material and Sample Preparation

Leaves of *H. speciosa* were collected in November 2015 in the municipality of Santa Rita, Paraíba State, Brazil. Identification of the plant species was validated by Professor Dr. Leonardo de Melo Versieux, from the Federal University of Rio Grande do Norte (UFRN), and a voucher specimen (UFRN20382) is currently deposited at the Herbarium of UFRN.

The collected material was air-dried in an air-forced oven at a continuous temperature of 40 °C for 72 h and ground in a mechanical mill prior to extraction. About 300 g of the powder were macerated in ethanol:water (70:30 *v*/*v*) at room temperature for 7 days under constant stirring. The suspension was filtered, and the solvent was removed under reduced pressure in a rotary evaporator at 40 °C. The extract was freeze-dried, and 10 mg were then homogenized with 1.5 mL of 100% methanol using an ultrasonic bath for 10 min. Extracts were then vortexed vigorously and centrifuged at 12,000 *g* for 5 min. For UHPLC-MS analyses, 1 mL of the supernatant was aliquoted and placed on a C18 cartridge (100 mg) preconditioned with water and methanol. The sample was then eluted using 1 mL of methanol and directly injected into the UHPLC system.

### 4.2. UHPLC-MS Analysis

The negative ion high-resolution ESI and collision-induced dissociation (CID) MS^n^ spectra were obtained from an Orbitrap Elite mass spectrometer (Thermo Fisher Scientific, Bremen, Germany) equipped with a heated electrospray ion source (negative spray voltage of 3 kV, capillary temperature of 300 °C, source heater temperature of 400 °C, Fourier Transform Mass Spectrometer (FTMS) resolution of 30.000). Nitrogen was used as a sheath and auxiliary gas. The MS system was coupled to a UHPLC system (Dionex UltiMate 3000, Thermo Fisher Scientific) equipped with a RP-18 column (particle size 1.9 µm, pore size 175 Å, 50 × 2.1 mm ID, Hypersil GOLD, Thermo Fisher Scientific; column temperature 40 °C) and a photodiode array detector (220–600 nm, Thermo Fisher Scientific). The mobile phases were H_2_O (A; Fluka Analytical, LC-MS Chromasolv) and CH_3_CN (B; Fluka Analytical, LC-MS Chromasolv) with 0.2% formic acid by using the following binary gradient at a flow rate of 400 μL/min: 0 to 1 min, isocratic 95% A, 5% B; 1 to 1.5 min, linear from 5% to 70% B; 1.5 to 8.5 min, linear from 70% to 100% B; 8.5 to 10 min, isocratic 100% B; and 10 to 15 min, isocratic 5% B. The injection volume was 2 μL. The CID mass spectra (buffer gas: helium) were recorded using normalized collision energy (NCE) of 35%. The instrument was externally calibrated by the Pierce ESI negative ion calibration solution (product No. 88324) from Thermo Fisher Scientific. The data were evaluated using the software Xcalibur 2.7 SP1.

### 4.3. Identification of Compounds in H. speciosa Extract via UHPLC-MS

The limit of detection (LOD) for every peak to be considered as caused by a compound was the signal-to-noise ratio (s/n) of 3:1. Compounds were characterized by their UV/Vis spectra (220–600 nm), retention time, full mass spectra, and MS^n^ fragmentation patterns, and by comparison to spectra of reference compounds and literature data. Accurate *m/z* values with their predicted molecular formula (error less than 5 ppm) were used for putative identification after database searches, such as in NIST, mass bank, Reaxys, SciFinder, and phytochemical dictionary database (CRC, Wiley). MS^2^ fragments were compared with compounds listed in the MassBank (http://www.massbank.jp/) or using in silico fragmentation in MetFrag (http://msbi.ipb-halle.de/MetFragBeta/). Identifications were confirmed with authentic compounds whenever available in-house.

## 5. Conclusions

The chemical characterization of the extract of *H. speciosa* leaves was achieved using a rapid and sensitive method—namely, UHPLC Orbitrap-HRMS. This technique allowed us to identify twenty-eight compounds, while just one remained unknown. From the identified compounds, seventeen were described for the first time for the plant species, of which the hydroxyl fatty acids found support for antibiotic claims, and the two phenylpropanoids phloretin and phlorizin and the flavonols quercetin and kaempferol support the proposed antidiabetic properties. The results demonstrate the potential of *H. speciosa* as a source of valuable phenolics. The knowledge obtained is of importance for future studies and possible uses of this plant, allowing a better analysis of cultivars or standardization of extracts. Further studies are needed to analyze the variation in the phenolic content of *H. speciosa* cultivated under distinct conditions, like regional, seasonal, and time of harvest influences.

## Figures and Tables

**Table 1:** Characterization of phenolic compounds and fatty acids in the leaves of *Hancornia speciosa* through UHPLC-HRMS/MS analysis in negative ionization mode.

Peak No.	[M − H]^−^ (*m/z*)	Rt (min)	UV (nm)	Molecular Formula	Error (ppm)	MS^n^ (*m/z*)	Suggested Compounds
1	191.0559	0.35	225, 263	C_7_H_11_O_6_	1.0	173, 155, 111, 87	Quinic Acid
2	315.0716	0.68	260, 369	C_13_H_16_O_9_	1.6	225, 153, 109	Protocatechuic acid *O*-hexoside
3 *	153.0193	0.85	264	C_7_H_6_O_4_	0	109	Protocatechuic acid
4	343.1027	1.30	264	C_15_H_20_O_9_	1.9	179, 135	Unknown
5	577.134	1.30	264	C_30_H_26_O_12_	1.8	451, 425, 407, 289	B-type Procyanidin
6 *	289.0712	1.64	298, 369	C_15_H_14_O_6_	1.8	245, 231, 205, 179	(+)-Catechin
7 *	353.0873	2.03	248, 323	C_16_H_18_O_9_	1.5	233, 191, 179, 173	Chlorogenic acid
8 *	179.0349	2.17	259, 370	C_9_H_8_O_4_	0.5	-	Caffeic Acid
9	577.1340	2.88	260, 370	C_30_H_26_O_12_	1.8	451, 425, 407, 289	B-type Procyanidin
10 *	289.0713	3.36	264, 369	C_15_H_14_O_6_	1.6	245, 231, 205, 179	(−)-Epicatechin
11	337.0922	3.45	298, 369	C_16_H_18_O_8_	1.7	191, 173, 163	3-*O*-(*E*)-p-Coumaroylquinic acid
12	865.1958	3.52	260, 370	C_45_H_38_O_18_	3.6	695,577	C-type Procyanidin
13	577.1340	3.56	260, 370	C_30_H_26_O_12_	1.8	451, 425, 407, 289	B-type Procyanidin
14	337.0924	3.62	298, 369	C_16_H_18_O_8_	1.5	191, 173, 163	3-*O*-(*Z*)-*p*-Coumaroylquinic acid
15 *	609.1453	3.65	285, 382	C_27_H_30_O_16_	1.3	591, 463, 447, 301	Rutin
16 *	463.0877	3.70	259, 353	C_21_H_20_O_12_	1.5	445, 301, 273, 178	Quercetin 3-*O*-hexoside
17	433.0770	3.72	269, 369	C_20_H_18_O_11_	1.1	151, 255, 300	Quercetin 3-*O*-pentoside
18	447.0927	3.74	266, 346	C_21_H_20_O_11_	1.4	429, 301, 151, 285	Quercetin 3-*O*-rhamnoside
19 *	435.1291	3.80	271, 340	C_21_H_24_O_10_	1.4	315, 273, 179	Phlorizin
20 *	273.0764	3.82	272, 350	C_15_H_14_O_5_	1.7	123, 125, 167, 179	Phloretin
21 *	287.0559	3.84	358	C_15_H_12_O_6_	0.7	135, 151, 169, 217	Eriodictyol
22 *	301.0351	3.93	269, 354	C_15_H_10_O_7_	0.9	273, 257, 178, 151	Quercetin
23 *	285.0402	3.93	299, 385	C_15_H_10_O_6_	1.0	133, 151, 175	Luteolin
24 *	269.0450	4.04	269, 336	C_15_H_10_O_5_	2.2	149, 159, 201, 225	Apigenin
25 *	285.0401	4.06	231, 360	C_15_H_10_O_6_	1.2	135, 151, 187	Kaempferol
26	327.2172	4.08	-	C_18_H_32_O_5_	1.8	227, 201, 171, 155	Trihydroxy-octadecadienoic acid
27	329.2326	4.15	-	C_18_H_34_O_5_	1.2	291, 209, 197, 155, 125	Trihydroxy-octadecenoic acid
28	311.2221	4.59	-	C_18_H_32_O_4_	1.2	-	Dihydroxy-octadecadienoic acid
29	293.2116	4.75	-	C_18_H_29_O_3_	1.8	275, 249	Hydroxy-octadecatrienoic acid

* Identification by co-injection with standards.
